# Occupational Exposure of Health-Care Workers to Blood and Body Fluids in West Shewa Zone, Ethiopia, 2018: A Cross-Sectional Study

**DOI:** 10.1155/2021/2944158

**Published:** 2021-12-31

**Authors:** Dejene Lemessa, Tesfaye Solomon

**Affiliations:** ^1^Oromia Regional Health Bureau, Addis Ababa, Ethiopia; ^2^Ethiopian Public Health Institute, Addis Ababa, Ethiopia

## Abstract

**Background:**

Health-care workers are susceptible to acquiring blood and body fluids borne infections due to their occupations involving contact with patients and their body fluids, although studies conducted in Ethiopia are scarce. Therefore, the aim of this study was to investigate the magnitude of exposure to blood and body fluids among health-care workers in governmental health facilities in West Shewa Zone, Ethiopia.

**Materials and Methods:**

A facility-based cross-sectional study was conducted from May 19 to June 25, 2018. A total of 381 health-care workers were selected by simple random sampling from 31 sampled governmental health facilities using proportional to size allocation. Data were collected through self-administered questionnaires, entered into Epi Info version 7, and analyzed by SPSS version 21. Adjusted odds ratio (AOR) with 95% confidence intervals (CI) calculated for variables retained in the multivariable logistic regression and significance declared at *p* < 0.05.

**Results:**

Of 377 health-care workers who participated, the study found that 233 (61.2%) were exposed to blood and body fluids in their lifetime. Previous needlestick injury (AOR = 0.30; 95% CI: 0.12–0.75), type of health facility (AOR = 0.42; 95% CI: 0.26–0.68), handwashing practice (AOR = 0.15; 95% CI: 0.07, 0.31), and perceiving at risk (AOR = 0.16; 95% CI: 0.03, 0.98) were protective factors, whereas long work experience (AOR = 1.47; 95% CI: 1.13–1.93) was a risk factor for the exposure.

**Conclusions:**

Exposures to blood and body fluids during patient care were common among health-care workers in the study area. Therefore, health-care workers especially those newly hired and working in hospitals should pay due attention to their occupation's safety and regularly practice handwashing during critical times.

## 1. Introduction

Health-care workers (HCW), whose occupations involve contact with patients and their body fluids, face a risk of exposure to occupational infections with a subsequent risk of contracting diseases, disability, and even death. HCW are constantly susceptible to acquiring blood-borne infections such as human immunodeficiency virus (HIV) and hepatitis [1, 2].

Occupational infections can be transmitted through inhalation, ingestion of contaminated material, and accidental needlestick inoculation [1]. Needlestick injuries are one of the most common methods of occupational transmission. HCW handling sharps such as scalpels and blood collection devices are also at risk of self-inoculating or needle-sticking wounds and subsequent exposure to blood-borne pathogens [3]. Occupational blood exposure can occur through percutaneous injuries (needlesticks or other sharp injuries), mucocutaneous (splashing of blood or other body fluids into the eyes, nose, or mouth), or contact with nonintact skin [4].

Health workers are exposed to a variety of hazards at work, including needlestick injuries, back injuries, latex allergy, violence, and stress [5]. The occupational risk of blood and body fluids (BBF) exposure and needlestick injuries affects not only the safety and well-being of HCWs but also the quality of medical care [6]. The risk of infection for health-care workers from blood-borne pathogens depends on the prevalence of these pathogens in the patient population and the type and frequency of exposure [7]. Most of these infections, however, can be prevented by practicing standard precautions, immunization against Hepatitis B, provision of personal protective equipment, and the management of exposure [4]. The transmission of HIV, hepatitis B virus (HBV), and hepatitis C virus (HCV) to patients by infected health-care workers has also been documented [8].

Standard precautions are based on the principle that all blood, body fluids, secretions, excretions except sweat, nonintact skin, and mucous membranes may contain transmissible infectious agents. Standard precautions include hand hygiene and, depending on the intended exposure, the use of gloves, gowns, masks, goggles, or face shields. It also includes equipment or items in the patient's environment that may have been contaminated with infectious fluid sand that must be handled to prevent transmission of infectious agents [9].

Of the 35 million health-care workers worldwide, the World Health Organization estimates that approximately 3 million percutaneous injuries are sustained each year. Of these injured health workers, 70,000 are likely to become infected as a result of exposure to the hepatitis B virus, 15,000 with HCV, and 1,000 with HIV [10], and 90% of the infections resulting from these exposures are carried by developing countries [11].

Exposure to needlestick injuries is higher in African countries than elsewhere and is a major public health concern due to overworked health workers. Similar studies in Ethiopia show that 32% of needlestick injuries were reported in the Sidama region, 31% in northwest Ethiopia, and 66% in 52 of the health facilities [12].

Few studies have been conducted in Ethiopia to assess the exposure status of blood and body fluids among health-care workers, and factors in health care facilities showed that the risk of exposure to BBF, particularly needlestick injuries, was widespread [13, 14]. The proportions of exposure to patients' body fluids differ significantly between different occupational groups [14].

Therefore, this study aimed to assess levels of exposure to blood and body fluids among health workers in government health facilities in the West Shewa Zone, Ethiopia. Hence, it provides information for program managers, practitioners, and decision-makers to focus and act on appropriate interventions. Additionally, it helps health workers know the severity and prevalence, minimize its impact, and be safe in their work environment.

## 2. Materials and Methods

### 2.1. Study Setting

The study was carried out in West Shewa Zone that is located in Oromia Regional State, Ethiopia. The zonal capital town, Ambo, is 112 km far to the west of the capital city, Addis Ababa. There are 22 districts in the zone. The zone has 1 referral, 3 general, and 3 primary hospitals and 91 health centers. Health center is the primary level of care in Ethiopia's health tier-system that has been serving around 25,000 in rural and 40,000 in urban catchment population with the aim of improving overall health outcomes, sanitation, maternal health, and infectious diseases. According to the West Shewa Zonal Health Office report [15], there are 1,959 health professionals and 821 supportive staff among the health facilities in the zone.

### 2.2. Study Design, Participants, and Period

A cross-sectional study design was used to assess levels of occupational exposure to BBFs and to identify associated factors among the sampled HCWs in selected governmental health care facilities in the West Shewa Zone. The government-employed health professionals such as pharmacists, environmental health professionals, and other support staff in the selected governmental health facilities were excluded because they were less likely to be exposed to blood and body fluids. The data were collected from May 19, 2018, to June 25, 2018.

### 2.3. Variables of the Study

Dependent variable was as follows: exposure to blood and body fluids.

Independent variables were as follows:

  Sociodemographic variables: age, sex, religion, ethnicity, educational status, current profession, and work experience
  Working environment: supply of personal protective equipment (PPE), guidelines/protocols, training, and number of patients attended daily
  Behavioral factors: use of PPE, recapping needles, and knowledge on occupational exposure

### 2.4. Sample Size Determination

The sample size was determined by using a single population proportion formula with the assumptions of a 95% confidence level, an error margin of 5%, and the prevalence of occupational exposure to BBFs (65.7%) [6]. Considering a 10% nonresponse rate, the final sample size was 381.

### 2.5. Sampling Procedure

A total of 99 governmental health facilities were divided into hospitals and health centers. Twenty-eight (30%) out of 91 health centers and 3 (33%) out of 9 hospitals were selected randomly. The sampled health professionals were then assigned in proportion to the total number of health care providers in each selected health facility. The register of health-care workers in selected governmental health facilities served as a sampling frame, and finally, a simple random sampling method was used to select health-care workers.

### 2.6. Data Collection Tools and Procedures

The data were collected through a self-administered questionnaire adapted from previous instruments used in various studies of exposure to blood and body fluids [6, 16–18]. The questionnaire has been grouped and arranged according to the specific objectives they can address. The questionnaire was originally prepared in English and translated into the local language, Afan Oromo. To check consistency, experts translated Afan Oromo's questionnaires back into English. A supervisor (BSC) was employed in each hospital. Four BSc supervisors were employed for the health centers (one supervisor: seven health centers).

### 2.7. Data Quality Assurance

The data collectors received one-day training on the objective, data collection process, and field ethics. Each questionnaire was checked daily by the supervisors and the principal investigator. The pretest was carried out at a nonsampled health center with 38 (5%) of the study. Due to the gaps found in the pretest, the necessary adjustments were made to the questionnaire. At the end of each day, the supervisors and investigators checked the questionnaire for completeness, correctness, and consistency and were discussed with all data collectors and supervisors.

### 2.8. Data Processing and Analysis

The collected data was cleaned, manually coded, and entered into EPI Info version 7 and exported to SPSS version 21 for analysis. A univariate analysis was done for continuous variables. A frequency distribution was carried out for categorical data. Bivariable analysis was performed to select candidate variables with *p* < 0.25. Then multivariate analysis was entered to identify independent predictor variables and control confounders. In the multivariate logistic regression, the adjusted odds ratio (AOR) with its 95% confidence intervals (CI) was computed for the variables retained in the final model, and the statistical significance was declared at *p* < 0.05.

### 2.9. Operational Definitions

#### 2.9.1. Health-Care Worker

Those people who do have contact with syringes, needles, other sharp materials, blood, and body fluids by virtue of their duties. Thus, nurses, laboratory technicians, physicians, dentists, health officers, X-ray technicians, cleaners, and laundry workers were considered.

#### 2.9.2. Exposure to Blood and Body Fluids

Exposure of health-care workers to blood or other body fluids such as semen, vaginal secretions, cerebrospinal, pleural, peritoneal, pericardial, and amniotic fluids via broken skin, eyes, mucous membranes, and sharp materials injury.

## 3. Results

### 3.1. Sociodemographic Characteristics

A total of 377 health-care workers participated in the study, yielding a response rate of 98.95%. The majority of the respondents 300 (79.58%) belonged to the age group 25 to 34 years. More than half of the participants, 225 (59.68%), were nurses by occupation (Table 1).

### 3.2. Environmental Factors of Exposure to Blood and Body Fluids

Of the 377 participants, 135 (35.81%) knew the availability of written guidelines for standard precautions. More than half of the respondents, 201 (53.32%) received HIV postexposure prophylaxis, while only 276 (73.21%) of the respondents received a hepatitis B vaccination. Of the respondents, only 49 (12.99%) took part in infection prevention or standard precaution training in the last year. Of the total participants, 268 (71.09%) mentioned there was a lack of personal protective equipment in their respective health facilities.

Among the total respondents, 208 (55.17%) stated that there are facilities for handwashing in their respective health facilities; however, only 78 (20.69%) of the respondents practiced handwashing after examining the patient. Of those, HCWs practicing handwashing before conducting any procedure was commonly practiced by nurses 43 (55.13%) followed by midwives 11 (14.10%). Of the total participants, 162 (42.97%) used personal protective equipment consistently. Among the PPE, the utility glove was the most frequently used 329 (87.27%), followed by the gown and the examination glove with 316 (83.82%) and 219 (58.09%), respectively.

### 3.3. Behavioral Factors and History of Exposure to Blood and Body Fluids

Of the total participants, 230 (61.01%) and 195 (51.72%) of health-care workers, respectively, have been exposed to BBFs and suffered needlestick injuries during their lifetime. Of 230 health-care workers exposed in the course of their lives, 174 (75.65%) had been exposed to blood at least once in the last year and 81 (21.49%) in the last 6 months. The second most common fluid to which workers were exposed was amniotic fluid 71 (18.83%), followed by breast milk 27 (7.16%). The majority of the exposed HCWs were 34 (14.78%) midwives. Performing multiple procedures simultaneously was the most common cause of exposure to blood and body fluids, 199 (52.79%; Table 2).

### 3.4. Factors Associated with Exposure to Blood and Body Fluids

A multivariate analysis was performed to determine independent predictors of exposure to blood and body fluids. Those candidate variables with *p* < 0.25 in the bivariate logistic regression were included in the multivariate logistic regression and considered significant in the model if the *p*-value was less than 0.05.

Health-care workers with less than or equal to 2 years of work experience were 1.47 times more likely to be exposed to blood and body fluids than those with 10 years of work experience (AOR = 1.47; 95% CI: 1.13, 1.93).

Health-care workers who worked in health centers were 58% less likely to be exposed to blood and body fluids than those who worked in hospitals (AOR = 0.42; 95% CI: 0.26, 0.68).

Health-care workers who were ever exposed to a needlestick injury during the procedure were 70% less likely to be exposed to blood and body fluids than those who were never exposed to a needlestick injury (AOR = 0.30; 95% CI: 0.12–0.75).

Pre- and postprocedure handwashing was an independent predictor of blood and body fluids exposure. Health-care workers who wash their hands before and after a procedure are 85% less likely to be exposed to blood and body fluids than those who do not wash hands (AOR = 0.15; 95% CI: 0.07, 0.31).

Health-care workers' perception of whether they are at risk for infections carried by blood and body fluid was found to be an associated factor in exposure to blood and body fluids. HCWs who were perceived as at risk were 85% times less likely to have had occupational exposure to blood and body fluids than those HCWs who did not consider themselves at risk (AOR = 0.16; 95% CI: 0.09–0.98; Table 3).

## 4. Discussion

This study was conducted to assess the magnitude of exposure to blood and body fluids in 377 participated health-care workers in governmental health facilities in the West Shewa Zone of central Ethiopia.

This study found that the lifetime exposure of health-care workers in government health care facilities to blood and body fluids was very high at 230 (61.2%), which exceeds reports from Harari and Dire Dawa (28.8%) [13], Georgia 53% [10], and Lebanon (30%) [19] but lower than a report from Wolaita (73.8%) and Gondar University Hospital (70.2%) [11, 16]. This suggests that health-care workers' exposure to blood and body fluids may be due to different routes of exposure and a lack of PPE in their health care facilities, which in turn may expose them to various body fluids and blood.

In this study, 81 (21.5%) of the study participants had been exposed to blood and body fluids in the last6 months, which is less than in the study in Wolaita 386 (62%) [11] and Bahir Dar Town 145 (45.7%) [6]. This difference may be due to the presence of safety signs in health facilities and the training of health workers in the workplace.

One hundred ninety-five (51.7%) of the study participants were injured by needlesticks in their lifetime, as this study shows, that is more than one study in Harari and Dire Dawa City, 145 (30.5%), and less than a finding in Nigeria 92 (53.5%) [20]. This suggests that health workers are at increased risk of work-related infections from blood and body fluids if effective measures are not taken.

In this study, 134 (35.5%) health-care workers were injured by needlesticks in the past year, which is much higher than the results from Awi Zone 36 (18.7%) and Bahir Dar Town 92 (29.0%) but less than a finding from Bale zone 126 (37.1%) [6, 21, 22]. This difference could be due to sociodemographic and economic differences in the study areas and the experience of health workers in following standard precautions.

This study found that health-care workers who were exposed to needlestick injuries were 70% less exposed to blood and body fluids compared to their counterparts, which could be due to the workers' care for themselves learning from the exposure to needlestick injuries.

This study found that the place in the level of health care system matter for occupational exposure to blood and body fluids. Health-care workers who work in the health center were 58% times less likely to be exposed to blood and body fluids compared to those who work in the hospital. This could be because health care providers who work in hospitals are more likely to come into contact with blood and body fluids from patients, are more involved in patient care activities, and have more contact with sharp instruments.

Health-care workers' perception of whether they were or not at-risk to body fluid and blood-borne infection was found to be an associated factor. HCWs who were perceived as being at risk of body fluid and blood-borne infection were 85% times less likely to have had occupational exposure to blood and body fluids than those who were perceived not at risk. Similar or different results have not yet been found in the literature reviewed. However, it appears to be due to the increased personal self-care through the use of personal protective equipment.

The year of service in the health system was also found to play a role in the occurrence of BBF exposure. In this study, health-care workers who had two or fewer years of work experience were 1.47 times more likely to be exposed to blood and body fluids as compared to health-care workers who had 10 or more years of job experience. This could be because young health workers may receive less infection prevention training as they stay in the system less.

Health-care workers who wash hands before and after each procedure were 85% less likely to be exposed to blood and body fluids than their counterparts. Since handwashing is a part of personal protection, these workers may be the ones who routinely take care of themselves. Washing hands after a procedure helps protect health-care workers from infection with patients' harmful germs and protect the health care environment from germ spread. Hence, health-care workers who wash their hands before and after a procedure might practice strong precautions against exposure to BBFs.

### 4.1. Limitation of the Study

The study relies on self-report rather than having a record review of health-care workers. Therefore, recall bias likely occurred as the information was obtained retrospectively.

## 5. Conclusions and Recommendations

Exposure to blood and body fluids during patient care was common among health-care workers in the study area. During their lifetime, 195 (51.7%) and 230 (61%) of the workers encountered needlestick injury and exposure to blood and body fluids, respectively. History of previous needlestick injury, place of work, and handwashing were protective factors, whereas long work experience was a risk factor associated with blood and body fluid exposure.

The health-care workers especially those newly hired and working in hospitals should pay due attention to their occupation's safety and regularly practice handwashing during critical times. The health facilities should avail and implement appropriate infection prevention protocols to reduce the risk of acquiring infection in health institutions.

## Figures and Tables

**Table 1:** Sociodemographic characteristics of health care workers, West Shewa zone, Ethiopia, 2018.

Variables	Category	Frequency	Percent
Sex	Male	186	49.34
	Female	191	50.66
Age in years	≤24	35	9.28
	25–34	300	79.58
	35–44	38	10.08
	≥45	4	1.06
Religion	Orthodox	167	44.30
	Protestant	153	40.58
	Catholic	3	0.80
	Muslim	30	7.96
	Waqeffata	24	6.37
Marital status	Single	145	38.46
	Married	226	59.95
	Separated	6	1.59
Educational status	Certificate	6	1.59
	Diploma	145	38.46
	Degree	223	59.15
	Specialist	3	0.80
Current profession	Nurse	225	59.68
	Laboratory	34	9.02
	Health officer	36	9.55
	Medical doctor	18	4.77
	Midwife	40	10.61
	Emergency and critical care nurse	24	6.37
Department of work	Out-patient department	90	23.87
	Injection and dressing room	39	10.34
	Surgical ward	20	5.310
	Pediatrics ward	23	6.10
	Gynecology ward	30	7.96
	Medical ward	20	5.31
	Laboratory	35	9.29
	Maternal and child health	120	31.83
Work experience	≤2 years	82	21.75
	3–5 years	147	39.00
	6–9 years	107	28.38
	≥10 years	41	10.87
Place of work	Health center	193	51.19
	Hospital	184	48.81

**Table 2:** Patterns of exposure to blood and body fluids among health care workers in West Shewa zone, Ethiopia, 2018.

Characteristics	Category	Exposure to blood and body fluids	
		Yes No. (%)	No No. (%)
Professional category	Nurse	145 (38.46)	80 (21.22)
	Laboratory	14 (3.71)	20 (5.31)
	Health officer	18 (4.77)	18 (4.77)
	Midwifery	34 (9.02)	6 (1.59)
	Medical doctor	9 (2.39)	9 (2.39)
	Others	10 (2.65)	14 (3.71)
Departments for occupational exposure	Inpatient	26 (6.90)	23 (6.10)
	Labor and delivery	193 (51.19)	110 (29.18)
	Laboratory	63 (16.72)	39 (10.34)
	Emergency	205 (54.38)	124 (32.89)
	Operating theatre	74 (19.63)	74 (19.63)
	Injection and dressing room	182 (48.28)	100 (26.53)
	Maternal and child health	1 (0.27)	1 (0.27)
Reasons for exposure	Multiple procedures simultaneously	199 (52.79)	122 (32.36)
	Sudden movement of the patient	54 (14.32)	44 (11.67)
	As a result of recapping of needle	102 (27.06)	50 (13.26)
	Lack of PPE	23 (6.10)	39 (10.34)
	During waste collection	46 (12.20)	11 (2.92)
	During delivery	19 (5.04)	4 (1.06)
	Not enough training on the issue	66 (17.51)	38 (10.08)
	Not following correct protocol	84 (22.28)	58 (15.38)
	Other	2 (0.53)	0 (0.00)

**Table 3:** Independent predictors of occupational exposure to blood and body fluids among health care workers in West Shewa zone, Ethiopia, 2018.

Characteristics	Category	Exposure to blood and body fluids		Crude OR (95% CI)	Adjusted OR (95% CI)
		Yes No. (%)	No No. (%)		
Work experience (in years)	≤2	35 (9.28)	47 (12.47)	0.15 (0.06–0.39)	1.47 (1.13–1.93)^*∗*^
	3–5	86 (22.81)	61 (16.18)	0.29 (0.12–0.69)	0.29 (0.12–0.69)
	6–9	75 (19.89)	32 (8.49)	0.48 (0.19–1.20)	0.48 (0.19–1.20)
	≥10	34 (9.02)	7 (1.86)	1	1
Place of work	Health center	143 (37.93)	50 (13.26)	3.19 (2.07–4.92)	0.42 (0.26–0.68)^*∗*^
	Hospital	87 (23.08)	97 (25.73)	1	1
Perceive at risk of BBF borne infection	Yes	223 (59.15)	121 (32.10)	6.85 (2.89–16.23)^*∗*^	0.16 (0.03–0.98)^*∗*^
	No	7 (1.86)	26 (6.90)	1	1
Ever injury of needle stick	Yes	154 (40.85)	41 (10.87)	5.24 (3.33–8.24)	0.30 (0.12–0.75)^*∗*^
	No	76 (20.16)	106 (28.12)	1	1
Washing of hands	Yes	32 (8.49)	46 (12.20)	0.29 (0.16–0.52)	0.15 (0.07–0.31)^*∗*^
	No	260 (68.97)	39 (10.34)	1	1

OR: odds ratio; CI: confidence intervals; and ^*∗*^*p* < 0.05 (significant association).

## Data Availability

The data used to support the finding of this study are included within the article.

## Abbreviations

AOR: Adjusted odds ratio
BBF: Blood and body fluid
CI: Confidence interval
HCW: Health-care workers
HBV: Hepatitis B virus
HCV: Hepatitis C virus
HIV: Human immunodeficiency virus
PPE: Personal protective equipment.
